# Enhancement of Carbon Conversion and Value-Added Compound Production in Heterotrophic *Chlorella vulgaris* Using Sweet Sorghum Extract

**DOI:** 10.3390/foods11172579

**Published:** 2022-08-25

**Authors:** Kangping Wu, Yilin Fang, Biyuan Hong, Yihui Cai, Honglei Xie, Yunpu Wang, Xian Cui, Zhigang Yu, Yuhuan Liu, Roger Ruan, Qi Zhang

**Affiliations:** 1 State Key Laboratory of Food Science and Technology, Engineering Research Center for Biomass Conversion, Ministry of Education, College of Food Science and Technology, Nanchang University, Nanchang 330047, China; 2 Queen Mary School, Jiangxi Medical College, Nanchang University, Nanchang 330047, China; 3 China Coal Zhejiang Testing Technology Co., Ltd., Hangzhou 310000, China; 4 Harbin Center for Disease Control and Prevention, Harbin 150000, China; 5 Australian Centre for Water and Environmental Biotechnology (Formerly AWMC), The University of Queensland, St. Lucia, Brisbane, QLD 4072, Australia; 6 Center for Biorefining, Department of Bioproducts and Biosystems Engineering and Department of Food Science and Nutrition, University of Minnesota, Saint Paul, MN 55108, USA

**Keywords:** *Chlorella vulgaris*, sweet sorghum, carbon source, biomass production, nutrient utilization

## Abstract

High-cost carbon sources are not economical or sustainable for the heterotrophic culture of *Chlorella vulgaris*. In order to reduce the cost, this study used sweet sorghum extract (SE) and its enzymatic hydrolysate (HSE) as alternative carbon sources for the heterotrophic culture of *Chlorella vulgaris*. Under the premise of the same total carbon concentration, the value-added product production performance of *Chlorella vulgaris* cultured in HSE (supplemented with nitrogen sources and minerals) was much better than that in the glucose medium. The conversion rate of the total organic carbon and the utilization rate of the total nitrogen were both improved in the HSE system. The biomass production and productivity using HSE reached 2.51 g/L and 0.42 g/L/d, respectively. The production of proteins and lipids using HSE reached 1.17 and 0.35 g/L, respectively, and the production of chlorophyll-a, carotenoid, and lutein using HSE reached 30.42, 10.99, and 0.88 mg/L, respectively. The medium cost using HSE decreased by 69.61% compared to glucose. This study proves the feasibility and practicability of using HSE as a carbon source for the low-cost heterotrophic culture of *Chlorella vulgaris*.

## 1. Introduction

Nowadays, the food supply in the world is facing considerable challenges due to the reduction of arable land caused by overpopulation [1,2]. The nutrient-rich nature of microalgae and their characteristic of not occupying any arable land make them an alternative for meeting global nutritional needs [3]. *Chlorella vulgaris* (*C. vulgaris*), which was first produced commercially at a large scale in Japan and Taiwan, has been added to drinks or food mainly as powders, capsules, and tablets [4,5,6]. It can provide the human body with nutritional supplements of biologically active compounds, such as polysaccharides, fatty acids, polyphenols, carotenoids, and so on. [7,8].

At present, the large-scale autotrophic culture of *C. vulgaris* mainly adopts the open pond culture system, which has a low yield and cannot meet the market demand [9,10]. In contrast, the heterotrophic cultivation of *C. vulgaris* in a closed fermenter using organic carbon sources can control the cultivation conditions, reduces the risk of microbial contamination, achieves higher biomass density and shorter culture periods, and promotes the growth of algal cells and the accumulation of specific products, such as proteins and lipids [11,12]. In addition, the high cell density obtained using a heterotrophic culture effectively reduces the cost of biomass harvesting [13].

The type of organic carbon source is considered to be one of the most momentous factors affecting the growth of microalgae under heterotrophic conditions [14]. Glucose is the most commonly used organic carbon source due to its superior culture performance compared to other substrates [15]. However, the high cost of glucose makes the heterotrophic culture of microalgae economically infeasible [16,17]. In recent years, scientists have used many economical substrates, such as acetic acid, algal residue, and sucrose, to replace glucose for microalgae cultivation [13,14,18]. However, the acquisition of such substrates also requires additional production. Pure substances (acetic acid and sucrose) need to be processed in a certain way, and algal residue is also a by-product of the lipid extraction process for algal biofuel production. Although the carbon conversion rate and the production of biodiesel are effective, other nutrients, except lipid components, have received less attention. Among many substrates, sweet sorghum (*Sorghum bicolor ‘Dochna’*) hydrolysate, which contains a lot of sugar, is a good option [19].

Sweet sorghum is a highly photosynthetic C4 plant with high biomass productivity and high amounts of fermentable sugar [20]. Due to its outstanding drought and salinity tolerance, sweet sorghum can be grown in marginal or semi-arid areas in western and northern China [21]. Sweet sorghum stems contain large amounts of soluble sugars, mainly sucrose, glucose, and fructose, which vary in content between varieties [22]. Therefore, sweet sorghum is very suitable for preparing the culture medium of heterotrophic *C. vulgaris* [23].

This study used enzymatic hydrolysates of sweet sorghum extract (HSE) instead of glucose for the heterotrophic cultivation of *C. vulgaris* to decrease the cost of carbon sources and increase the production of higher-value metabolites. Firstly, sucrase was used to hydrolyze the sucrose in the sweet sorghum extract (SE) into glucose and fructose. Then, SE and HSE supplemented with nitrogen and minerals were used as the media for the heterotrophic culture of *C. vulgaris*. This study provides a new strategy for the heterotrophic culture of *C. vulgaris* by using HSE instead of glucose, which reduces the cost of the culture medium and increases the value of the product.

## 2. Materials and Methods

### 2.1. Materials

*Chlorella vulgaris* (FACHB-32) was obtained from the Institute of Hydrobiology, at the Chinese Academy of Sciences, P. R. Sweet sorghum seeds were purchased from Hebei Lihong Seed Industry Co., Ltd. and planted at the Engineering Research Center for Biomass Conversion of Nanchang University. Sweet sorghum stalks were obtained by manual defoliation after harvest. Then, the sweet sorghum stalks were squeezed with a juicer at a weight ratio of stalk-to-water of 1:1.5 and filtered with double-layer gauze to obtain the SE. The obtained SE was stored in a refrigerator at 4 °C for further use. All chemicals used in this study were of analytical grade. The sucrase used to hydrolyze the SE was purchased from Guangzhou Tianjin Food Additive Co., Ltd. Its optimum enzymatic hydrolysis temperature is 45–50 °C, its optimum enzymatic hydrolysis pH is 4.0–4.5, and its enzyme activity is 100,000 U.

### 2.2. Preparation of HSE

The HSE was obtained by adding 0.10% sucrase, and it was maintained at 50 °C for 4 h in a vortex. The hydrolysate was then boiled for 10 min to inactivate the enzymes. After cooling, it was filtered with a 0.45 µm-micron membrane to obtain HSE. The obtained HSE was stored at 4 °C for later use.

### 2.3. Experimental Design

The compositions of the media for the heterotrophic culture of *C. vulgaris* are shown in Table 1 in detail. The BG-11 medium (BG-11+glu) containing 10 g/L glucose was used as the basic medium for the heterotrophic culture of *C. vulgaris*. When the SE and HSE were used as carbon sources, the total carbon content in the medium was controlled to be consistent with the BG-11+glu medium. HSE medium, HSE+N medium (HSE supplied with nitrogen source equivalent to BG-11), HSE+M medium (HSE supplied with minerals equivalent to BG-11), HSE+N+M medium (HSE supplied with nitrogen source and minerals equivalent to BG-11), SE+N+M medium (SE supplied with a nitrogen source and minerals equivalent to BG-11) were used for the heterotrophic culture of *C. vulgaris* to study whether SE and HSE could provide a carbon source, nitrogen source, and minerals for the growth of microalgae. The seed solution of *C. vulgaris* was inoculated in a BG-11 medium containing 10 g/L glucose for the heterotrophic culture. Before the formal culture inoculation, the inoculant was transferred to a sterile centrifuge tube, centrifuged at 10,000 rpm at 4 °C for 5 min, washed twice with sterile deionized water, and resuspended with a certain amount of sterile water. Then it was inoculated into 300 mL of the above sterile medium in a sterilized 500 mL Erlenmeyer (121 °C, 20 min) flask, and the inoculation concentration was 0.05 g/L. The cultures were placed in a shaker and kept in a dark environment of 28 °C with a shaking speed of 120 rpm for 6 days. During the culture process, 6 mL of the culture was taken from each flask every 24 h for subsequent analysis. The inoculation and sampling processes were carried out in a sterile room. After the cultivation, the remaining microalgae liquid was collected and centrifuged at 8000 rpm for 10 min. The supernatant was discarded. The microalgae sludge was washed twice with deionized water, freeze-dried in a large petri dish, and stored at −20 °C. All the experiments were carried out in triplicate.

### 2.4. Analytical Methods

#### 2.4.1. Microalgae Biomass and pH

The biomass concentration was determined gravimetrically. Briefly, after centrifugation at 8000 rpm for 5 min at 4 °C, the microalgal cell pellets were collected and washed twice with distilled water, then dried at 80 °C for 24 h [24]. The growth kinetic parameters of microalgal cells were calculated according to the method of Cai et al. [25]. The specific growth rate per day (*μ*) of the *C. vulgaris* was calculated according to Equation (1):

(1) $$\mu =\left(\ln W_{n}-\ln W_{n-1}\right)$$

where *W*_*n*_ and *W*_*n*−1_ are the dry cell weights on day *n* and day *n* − 1, respectively.

The generation time of the *C. vulgaris* was calculated according to Equation (2):

(2) $$\mathrm{Generation}\;\mathrm{time}\;(d)=\left(\ln2\right)/\left(\mu_{\max}\right)$$

The biomass productivity of the *C. vulgaris* was calculated according to Equation (3):

(3) $$\mathrm{Biomass}\;\mathrm{productivity}\;(g/L/d)=\left(M_{2}-M_{1}\right)/6$$

where *M*_1_ and *M*_2_ are the biomass concentration at the beginning and the end of the culture, respectively.

The growth yield of the *C. vulgaris* was calculated according to Equation (4):

(4) $$\mathrm{Growth}\;\mathrm{yeild}\;(g/g)=\left(M_{2}-M_{1}\right)/\left(N_{2}-N_{1}\right)$$

where *M*_1_ and *M*_2_ are the biomass concentrations at the beginning and the end of the culture, respectively. *N*_1_ and *N*_2_ are the total organic carbon concentrations at the beginning and the end of the culture, respectively.

The pH of the culture medium was daily monitored using a Raymagnet pH composite electrode (E-201-C, Inesa, Shanghai, China).

#### 2.4.2. Nutrient Analysis of the Medium

The algal fluid samples obtained daily were centrifuged at 8000 rpm and for 5 min at 4 °C. Then the supernatant was filtered through a 0.45 µm-micron membrane for nutrient analysis. Since glucose and fructose are isomers that can be converted into each other through isomerization under alkaline conditions, the reducing sugar (including glucose and fructose) concentration in the supernatant was determined using the 3,5-dinitrosalicylic acid (DNS) method under alkaline conditions according to the method of Wang et al. [13]. The diluted supernatant (1 mL) was mixed with 1.5 mL of DNS, and then stored at 100 °C for 5 min. After cooling, the volume of the sample was adjusted to 25 mL, and its absorption at a wavelength of 540 nm was detected using a UV/visible spectrophotometer (UV-9000, Metash, Shanghai, China). The fructose concentration in the supernatant was determined using fructose test kits (Jiancheng, Nanjing, China). The concentrations of the total organic carbon (TOC), total inorganic carbon (IC), and total nitrogen (TN) in the supernatant were determined by the internal standard method using a multi-N/C 3100 analyzer (Analytik Jena Ag, Jena, Germany) according to the method of Gu et al. [26]. The metal elements (Cu^2+^, Zn^2+^, Mg^2+^, K^+^, Ca^2+^, Cu^2+^, Fe^3+^, and Mn^2+^) in SE and HSE were determined using an ICP-MS (7800, Agilent Technologies, Lake Forest, CA, USA) according to the method of Huang et al. [27]. The SE and HSE were mixed with concentrated nitric acid (1:2, *v*/*v*) and further digested in a microwave digestion system (MDS-6g, SINEO, Shanghai, China) under the conditions of 150 °C for 10 min and 180 °C for 15 min. After digestion, the samples were cooled to room temperature and diluted to 50 mL with ultrapure water. Then, the samples with a certain dilution were determined by the internal standard method using an ICP-MS (7800, Agilent Technologies, Lake Forest, CA, USA).

#### 2.4.3. Chemical Composition Analysis of the Microalgae

The chlorophyll-a and carotenoid extracted by methanol were measured by s UV/visible spectrophotometer (UV-9000, Metash, Shanghai, China) according to the method of Zhou et al. [28]. The algal sludge remaining after centrifugation in Section 2.4.2 was washed three times with distilled water, and the supernatant was discarded. Then, 99.80% (*v*/*v*) methanol was added to the algal mud to mix evenly and incubated in the dark at 4 °C for 24 h. After centrifugation, the absorbance of the supernatant at 665.2, 652.4, and 470 nm was assayed using s UV/visible spectrophotometer (UV-9000, Metash, Shanghai, China). Then, the concentrations of chlorophyll-a (chl-a) and carotenoid were calculated according to Equations (5) and (6):

(5) $$\mathrm{Chl}-a\;(\mathrm{mg}/L)=16.72\times A_{665.2}-9.16\times A_{652.4}$$

(6) $$\mathrm{Carotenoid}\;(\mathrm{mg}/L)=\left(1000\times A_{470}-1.63\times \mathrm{Chl}-a\right)/221$$

where *A*_665.2_*, A*_652.4_, and *A*_470_ are the absorbance values at 665.2, 652.4, and 470 nm respectively.

The lutein was determined by the external standard method using high-performance liquid chromatography (1260, Agilent Technologies, Lake Forest, CA, USA) according to the method of Solovchenko et al. [29]. The 30 mg freeze-dried microalgal powder added with 3 mL of dimethyl sulfoxide was subjected to ultrasonication and then reacted at 70 °C for 2 h until the powder became white. Then it was analyzed by high-performance liquid chromatography after passing through a 0.22 µm-micron membrane. The HPLC conditions were as follows: separation column: Eclipse Plus C18 (250 mm × 4.6 mm, 5 μm); mobile phase: tetrahydrofuran/methanol/acetonitrile (5:45:50, *V*/*V*/*V*); detector: UV detector; detection wavelength: 446 nm; column temperature: 30 °C; flow rate: 1.0 mL/min. The protein was determined using an automatic Kjeldahl analyzer (K9860, Manon, Dezhou, China) according to the method of Xie et al. [30]. Firstly, 10 mL of nitric acid was added to 0.1 g of algal flour and 0.5 g of Kjeldahl catalyst sheet (potassium sulfate/copper sulfate = 15:1). The mixture was digested in a graphite digester (SH620DF, Manon, Dezhou, China). The digestion conditions were, first, 180 °C for 50 min, then 280 °C for 20 min, and finally, 420 °C for 80 min. After cooling, the samples were subjected to protein determination using an automatic Kjeldahl analyzer (K9860, Manon, Dezhou, China). The lipid content in the microalgae powder was determined by the method of He et al. [31]. Firstly, 0.1 g of algal powder (the mass is recorded as *X_1_*) was added with 3 mL of chloroform/methanol (2:1, *V*/*V*). After sonication, the mixture was centrifuged at 8000 rpm, 4 °C for 10 min, and the supernatant was collected. Then, 1 mL of 0.90% sodium chloride solution was added to the collected solution. After sufficiently shaking and standing, the volume of the low-phase solution was measured and recorded as *V*. Then, 5 mL of a low-phase solution was added to a pre-weighed centrifuge tube (weighed as *X*_0_) and weighed as *X*_1_ after drying with nitrogen. The lipid content was calculated according to Equation (7):

(7) $$\mathrm{Lipid}\;(g/g)=\left(X_{2}-X_{0}\right)\times V/\left(5\times X_{1}\right)$$

#### 2.4.4. Statistical Analysis

The experiments were done in triplicate, and the results were expressed in the form of mean ± standard deviation (n = 3). Microsoft Excel 2016 and IBM SPSS Statistic 23 were used for statistical analysis of the data, and Origin 2019b was used for data plotting. One-way ANOVA analysis of variance and Duncan test were used to analyze the mean and standard deviation values. A *p* < 0.05 is a significant difference.

## 3. Results and Discussion

### 3.1. Basic Compositions of SE and HSE

The main sugars in the stems of sweet sorghum are sucrose, fructose, and glucose [22]. During the enzymatic hydrolysis of sweet sorghum extract, the sucrose was hydrolyzed into glucose and fructose. The components of SE and HSE are shown in Table 2. The contents of the reducing sugar, fructose, TOC, IC, and TN in the SE were 56.75, 31.28, 32.05, 0.34, and 0.18 g/L, respectively. The contents of the reducing sugar, fructose, TOC, IC, and TN in the HSE were 86.69, 42.25, 34.04, 0.17, and 0.37 g/L, respectively. Compared to SE, the contents of the reducing sugar and fructose in the HSE increased by 29.94 and 10.97 g/L, respectively, due to the decomposition of the sucrose. The contents of TOC and TN in the HSE increased by 1.99 and 0.19 g/L, respectively, compared to the SE, due to the introduction of organic carbon and nitrogen source by sucrase. Studies have reported that sweet sorghum contains many trace mineral elements, such as Mg, K, Ca, Cu, Mn, Fe, Zn, etc. [32,33]. The contents of K, Ca, and Mg in sweet sorghum stems are higher, while other mineral elements have trace concentrations [33]. In this study, only the mineral ions Mg^2+^, K^+^, and Ca^2+^ in the SE were detected by the ICP-MS. The concentrations of Mg^2+^, K^+^, and Ca^2+^ in SE were 62.15, 632.30, and 177.75 mg/L, respectively. Other trace mineral ions were not detected by the ICP-MS (the detection line of trace mineral ions was 0.001 mg/L). There was little difference in the mineral ions concentrations in the HSE, the concentrations of Mg^2+^, K^+^, and Ca^2+^ were 61.40, 658.40, and 170.25 mg/L, respectively. There was no significant difference in the mineral ion concentrations before and after hydrolysis (*p* < 0.05). These mineral elements and other soluble organic matters in sweet sorghum extract might be suitable nutrients for *C. vulgaris*.

### 3.2. Changes in Biomass and Organic Carbon during the Cultivation of Chlorella Vulgaris

**Figure 1:**
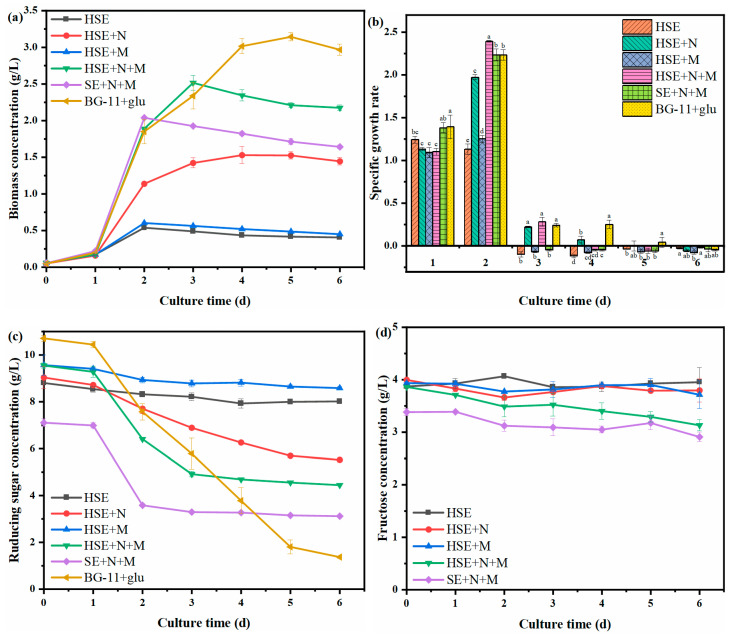

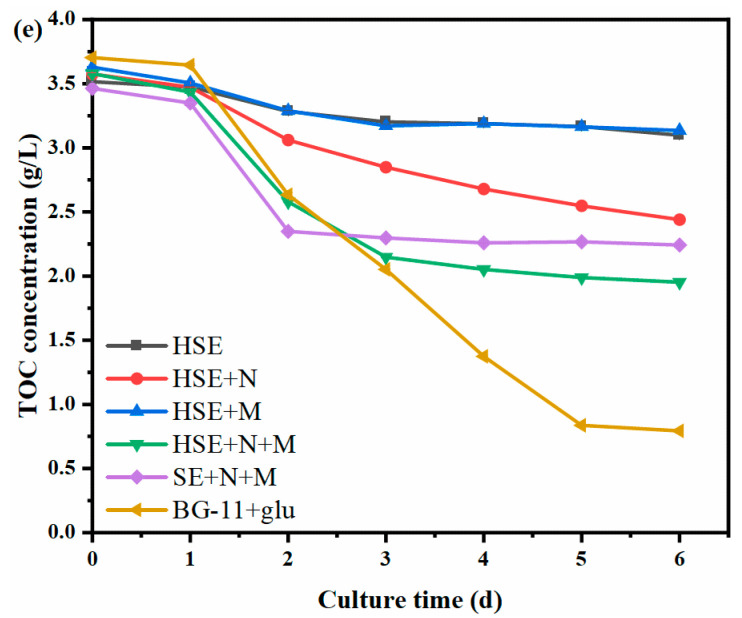
The growth curves of heterotrophic *C. vulgaris* (**a**), the specific growth rate of heterotrophic *C. vulgaris* (**b**), the reducing sugar concentration curves (**c**), the fructose concentration curves (**d**), and the total organic carbon concentration curves (**e**) during the culture of heterotrophic *C. vulgaris*. Different superscripts (a–e) indicate significant differences (*p* < 0.05).

The changes in the biomass production of *C. vulgaris* are shown in Figure 1a, and the growth kinetic parameters are shown in Table 3. *C. vulgaris* in all groups experienced a lag phase in the first 24 h. After 24 h, the cells adapted to the medium environment, and the *C. vulgaris* entered the exponential growth phase. The biomass of each group decreased slowly after reaching the highest value. The HSE, HSE+M, and SE+N+M groups reached the highest biomasses after 2 days of culture, reaching 0.54, 0.60, and 2.04 g/L respectively. The HSE+N+M, HSE+N, and BG-11+glu groups reached the highest biomasses of 2.52, 1.53, and 3.14g/L in the following days, respectively. The maximum biomass productivity was significantly different among different groups (*p* < 0.05), and these differences might be mainly due to the differences in the composition and contents of sugar among the groups of HSE, SE, and BG-11+glu. Zhang et al. [34] found that it was difficult for *C. vulgaris* to effectively utilize sucrose. In order to further clarify the difference in the biomass of *C. vulgaris*, the main monosaccharide contents together with the TOC in the medium were also measured in this study.

The specific growth rate and the changing curves of the reducing sugar concentration are shown in Figure 1b,c. In the first 24 h, *C. vulgaris* was in the lag phase, and the reducing sugar content decreased slowly (each group only decreased by about 0.2 g/L). After 24 h, there were differences in the reducing sugar consumption among the groups, and significant differences in the growth rate appeared among different groups. The reducing sugars in the HSE and HSE+M groups without a nitrogen source were difficult to be used by *C. vulgaris*; the rates of consumption of the reducing sugar in these two groups were only 0.79 and 0.98 g/L, respectively. At the end of the culture, the biomass concentration and productivity were both very low. The reason for this phenomenon might be that the small amount of nitrogen source contained in the sweet sorghum extract could meet the needs of the early growth of *C. vulgaris*, while the growth of *C. vulgaris* in the treatment without the nitrogen source supplement was inhibited after the exhaust of the contained nitrogen source. This result indicates that a sufficient nitrogen source is a necessary factor for the normal growth of *C. vulgaris*. Compared to the HSE and HSE+M groups, *C. vulgaris* in the HSE+N group could continue to grow, and the group consumed a total of 3.51 g/L glucose during the culture. However, its biomass was still inferior to the group supplemented with nitrogen sources and minerals. The highest biomass concentration and productivity of the HSE+N group were 1.53 g/L and 0.25 g/L/d, which were 60.96% and 59.52% of those of the HSE+N+M group. This indicates that the minerals in the extract of sweet sorghum could not support the massive growth of *C. vulgaris*. The reducing sugar concentration of the SE+N+M and HSE+N+M groups decreased by 3.70 g/L and 4.36g/L, respectively, from the first day to the third day. Apparently, the *C. vulgaris* in the SE+N+M and HSE+N+M groups could rapidly absorb and utilize reducing sugars, resulting in a rapid increase in biomass. However, the reducing sugar concentration of the above two groups gradually stabilized on day 3, and the biomass of *C. vulgaris* no longer increased. As can be seen from Figure 1d, the fructose concentration in the medium remained basically unchanged. This indicates that it was difficult for the *C. vulgaris* to effectively utilize the fructose during the heterotrophic culture. Therefore, the remaining unutilized reducing sugars in the SE+N+M medium and HSE+N+M medium were fructose. The maximum biomass concentration and productivity of *C. vulgaris* in the SE+N+M group were 2.04 g/L and 0.34 g/L/d, which were 64.97% and 65.38% of those of the BG-11+glu group (3.14 g/L and 0.52 g/L/d). The maximum biomass concentration and productivity of *C. vulgaris* in the HSE+N+M group were 2.51 g/L and 0.42 g/L/d, respectively, which were 79.94% and 80.77% of those of the BG-11+glu group. Although the maximum biomass concentration and productivity were slightly lower than those in the BG-11+glu group, the growth yield (g biomass/g TOC) of the *C. vulgaris* in both the SE+N+M and the HSE+N+M groups were 1.30 times that of the BG-11+glu group (Table 3). The results show that the conversion efficiency rates of the carbon sources to the biomass of *C. vulgaris* in the media of SE and HSE were higher than that in the pure glucose medium under the conditions of sufficient nitrogen sources and minerals. The *C. vulgaris* in the SE+N+M and HSE+N+M groups reduced the waste of organic carbon and converted more organic carbon into biomass. The change curves of the TOC concentration are shown in Figure 1e. The levels of organic carbon in each group remained at the same level at the beginning, and the decreasing trend of organic carbon was roughly consistent with the decreasing trend of reducing sugar. The total organic carbon concentrations in the HSE, HSE+N, HSE+M, HSE+N+M, SE+N+M, and BG-11+glu groups decreased by 0.42, 1.14, 0.49, 1.63, 1.22, and 2.91 g/L, respectively, over the entire culture period. However, the final organic carbon level in the SE+N+M medium (2.24 g/L) was higher than that in the HSE+N+M medium (1.95 g/L) due to the large amount of non-enzymatic sucrose in it.

### 3.3. Changes in the pH and Total Nitrogen during the Cultivation of Chlorella vulgaris

**Figure 2:**
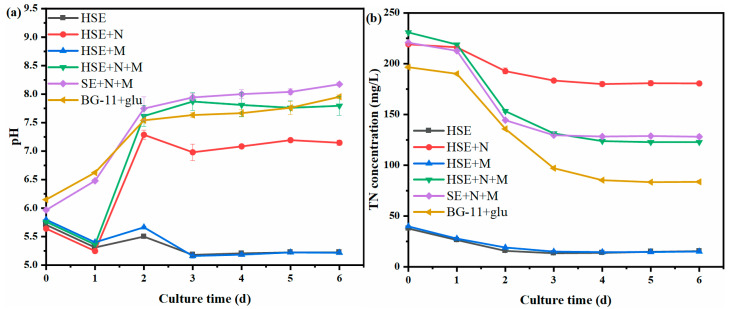
The change curves of pH (**a**) and the total nitrogen concentration (**b**) during the culture of heterotrophic *C. vulgaris*.

The pH is one of the most critical factors affecting the growth and metabolism of microalgae [35]. The pH of the culture medium was monitored during the cultivation of *C. vulgaris*, and the results are shown in Figure 2a. The initial pH of each experimental group was around 5.80. During the culture process, the pH of the HSE and HSE+M groups without a nitrogen source was maintained within a certain range after dropping to about 5.30 in the first 24 h. The pH of the HSE+N and HSE+N+M groups added with a nitrogen source both decreased to about 5.30 and then increased to 7.20 and 7.60 in the first 48 h, respectively. However, the pH of the SE+N+M and BG-11+glu groups did not drop. The pH of the two groups continued to rise from around 6 and finally stabilized at around 8. The reason for this change was the absorption and utilization of ammonium nitrogen by the *C. vulgaris*. If *C. vulgaris* assimilates ammonium nitrogen, hydrogen ions will be released into the medium during the process, resulting in a decrease in the pH; if it assimilates nitrate nitrogen, hydroxide ions will be released, resulting in an increase in the pH [36,37]. In the presence of both ammonium nitrogen and nitrate nitrogen, the nitrate reductase in *C. vulgaris* cells is inhibited by the ammonium nitrogen [36]. On the other hand, less energy is required to utilize ammonium nitrogen [38,39]. Therefore, in this case, *C. vulgaris* will preferentially utilize ammonium nitrogen, and as the concentration of ammonium nitrogen decreases, *C. vulgaris* will begin to utilize nitrate nitrogen [25]. In this study, sucrase was used in the enzymatic hydrolysis of the sweet sorghum extract. In the production process, the sucrase needs to be precipitated with ammonium sulfate. These incompletely scavenged ammonium sulfates were introduced into the medium, resulting in the presence of a small amount of ammonium nitrogen in the HSE. The *C. vulgaris* in all HSE groups preferentially utilized this part of the ammonium nitrogen, which led to a decrease in the pH of the medium in the first 24 h. After that, the *C. vulgaris* utilized the nitrate nitrogen, resulting in a dramatic rise in pH. This was also confirmed by the measurement of the total nitrogen in the SE and HSE prior to the medium configuration (Section 3.1). Yadav and Singh [40] found that a slightly alkaline pH (7.50–8.50) environment was optimal for the synthesis of proteins and carbohydrates by *C. vulgaris*. In addition, a weakly acidic pH (6.00) environment was conducive to the absorption and utilization of glucose in the medium by *C. vulgaris* [25]. Under the culture strategy of this study, the *C. vulgaris* efficiently utilized the glucose under acidic conditions in the early stage, and then entered a weak alkaline environment that was conducive to the synthesis of value-added chemicals.

The change curves of the TN concentration are shown in Figure 2b. The initial nitrogen concentration of the HSE and HSE+M groups without a nitrogen source were both about 38.00 mg/L, and they were consumed by about 23.00 mg/L at the end of the experiment. These two groups had the lowest biomass productivity (Table 3). The data reconfirm that a sufficient nitrogen source was a necessary factor for the normal growth of *C. vulgaris*. Studies have shown that when the nitrogen concentration was too low, the microalgae were stressed and growth was not apparent [41,42]. Meanwhile, when the *C. vulgaris* was in a nitrogen-rich environment, the TN concentration of the HSE+N, HSE+N+M, SE+N+M, and BG-11+glu groups decreased by 39.00 mg/L, 108.00 mg/L, 92.60 mg/L, and 112.90 mg/L, respectively. This fully indicates that minerals were also an important factor affecting the growth and metabolism of *C. vulgaris*, and the minerals that the SE and HSE contained were not sufficient for the heterotrophic growth of *C. vulgaris*.

### 3.4. Chemical Composition of Chlorella vulgaris

**Figure 3:**
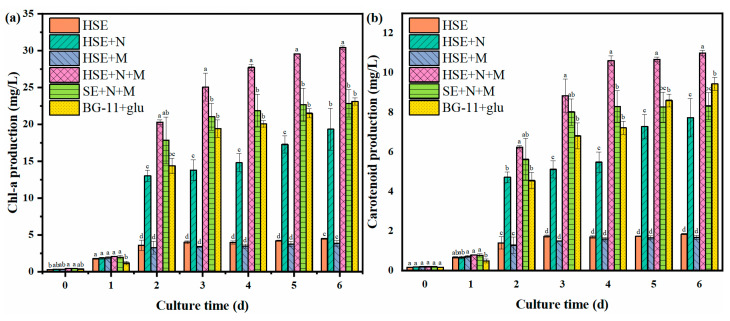

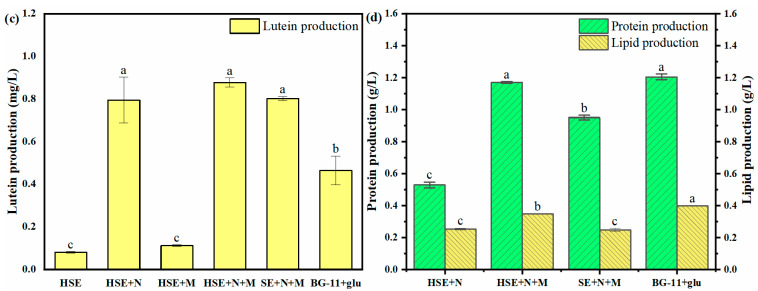
The productions of chlorophyll-a (**a**), carotenoid (**b**), lutein (**c**), protein, and lipid (**d**) of the harvested heterotrophic *C. vulgaris*. Different superscripts (**a**–**d**) indicate significant differences (*p* < 0.05).

In this study, the value-added compounds of *C. vulgaris* were analyzed to evaluate the nutritional value of microalgae. The chlorophyll-a and carotenoid production were monitored during the culture process, and the results are shown in Figure 3a,b. The results show that the production of chlorophyll-a and carotenoids in each group accumulated and increased with time. At the end of the culture, the HSE+N+M group had the highest chlorophyll-a and carotenoid production, reaching 30.42 mg/L and 10.99 mg/L, respectively, which were rates 1.3 and 1.2 times higher than those of the BG-11+glu group. The contents of chlorophyll-a and carotenoid of the *C. vulgaris* in the HSE+N+M group reached 13.99 mg/g and 5.05 mg/g, which were rates 1.8 times and 1.6 times higher than those in the BG-11+glu group (Table 4). There were no significant differences in the productions of chlorophyll-a and carotenoid between the SE+N+M and BG-11+glu groups. However, the chlorophyll-a and carotenoid contents of the *C. vulgaris* in the SE+N+M group were 13.90 mg/g and 5.07 mg/g, which were rates 1.8 times and 1.6 times higher than those of the BG-11+glu group (Table 4). The results show that both the SE and HSE medium were beneficial for the *C. vulgaris* to accumulate the chlorophyll-a and carotenoid products. Mineral ions Mg^2+^ and Fe^2+^ are essential for the chlorophyll function of the microalgae as cofactors in the various metabolic pathways of microorganisms [43]. The chlorophyll-a production in the *C. vulgaris* was enhanced due to the presence of these ions, especially magnesium ions, in the SE and HSE. As a carotenoid, lutein has antioxidant, anti-inflammatory, and other functional properties [44]. The lutein production of *C. vulgaris* is shown in Figure 3c. The lutein production of *C. vulgaris* in the BG-11+glu medium was only 0.47 mg/L. However, the lutein production was significantly increased in several groups with a nitrogen source added. The lutein production was 0.80 mg/L in the HSE+N group, 0.88 mg/L in the HSE+N+M group, and 0.80 mg/L in the SE+N+M groups, which were rates 1.7, 1.9, and 1.7 times higher, respectively, than those of the BG-11+glu group. Obviously, the *C. vulgaris* in these groups had significantly higher lutein contents (Table 4). Carotenoids (including lutein) are functional antioxidant pigments accumulated by *C. vulgaris* under stressful adversity [45]. However, in the SE and HSE, carotenoids of *C. vulgaris* were accumulated, and the mechanism is still unclear. When some scholars studied the use of sweet sorghum juice to cultivate yeast, there were also unexplained phenomena [46]. The authors speculated that there was a class of inhibitory compounds affecting the yeast in the sweet sorghum juice. Such inhibitory compounds may be responsible for the accumulation of carotenoids in *C. vulgaris*. The protein and lipid productions of each group with a nitrogen source added are shown in Figure 3d. The amount of *C. vulgaris* harvested in the HSE and HSE+M groups was insufficient due to the low biomass production. Therefore, the protein and lipid contents of these two groups were not measured. The results show that the protein productions of HSE+N+M and BG-11+glu groups were both around 1.20 g/L, and there was no significant difference (*p* < 0.05). However, the *C. vulgaris* in both the HSE+N+M and SE+N+M groups were higher in terms of the protein content (0.54 g/g and 0.58 g/g), which were rates 1.3 times and 1.4 times higher than those of the BG-11 group (Table 4). The nitrogen consumption of *C. vulgaris* in the HSE+N+M and SE+N+M groups was comparable to that of the BG-11+glu group, while those two groups had lower biomass productions (Section 3.2 and Section 3.3). This indicates that the utilization rate of the nitrogen source by *C. vulgaris* was improved in the SE and HSE. The lipid content of the HSE+N+M group was 0.16 g/g, which was 1.2 times that of the BG-11+glu group (Table 4). However, its lipid production was lower than that of the BG-11+glu group due to its lower biomass. 

In conclusion, in the HSE+N+M group, the pigment production was higher, the protein production was comparable, and the lipid production was slightly lower, compared to the BG-11+glu group. However, the contents of the above compounds in the HSE+N+M group were significantly higher (*p* < 0.05) than that of the BG-11 group. Chlorophyll-a, carotenoids, and lutein are all beneficial to human health. The *C. vulgaris* cultivated in this study had higher nutritional value as food, which was beneficial to human health.

### 3.5. Cost Assessment

In this study, the applicability of HSE to replace glucose as a carbon source in the heterotrophic culture of *C. vulgaris* was evaluated through an economic analysis of the medium cost. The medium cost per kilogram of biomass was calculated according to the medium input and the biomass output ratio in this study. In Table 5, the results show that the cost of the carbon source is the bulk of the medium cost in the BG-11+glu medium. The glucose cost (USD 2.411) per kilogram of biomass accounted for 87.86% of the total cost (USD 2.744). In contrast, the biomass cost per kilogram of the HSE+N+M group was only USD 0.834, which was 30.39% of the BG-11+glu group. The cost of the SE+N+M group was USD 0.672 per kilogram of biomass, which was 24.49% of that of the BG-11+glu group. The economic benefits of using sweet sorghum as a carbon source to cultivate *C. vulgaris* are outstanding. Therefore, this cultivation strategy could effectively enhance the production of C. vulgaris and reduce the costs by using the sweet sorghum extract. 

Additionally, the culture strategy employed in this study was sterile and safe. The microalgae were finally obtained and the remaining medium could both be used as food raw materials. In this study, although the glucose was basically consumed, the fructose and some minerals that could not be absorbed and utilized by *C. vulgaris* remained in the medium. These residual components could well be utilized for the production of sweeteners and minerals for human foods. Future work of this study will be focused on the production of functional beverages by using the harvested *C. vulgaris* together with the residual culture medium.

## 4. Conclusions

Hydrolysates of sweet sorghum extract could provide a carbon source for the growth of *Chlorella vulgaris*. After supplementing with nitrogen sources and mineral elements, *Chlorella vulgaris* in the hydrolysates of sweet sorghum extract could consume nitrate nitrogen to increase the pH and become conducive to value-added compound synthesis. The hydrolysates of sweet sorghum could not only ensure high biomass but also enhance the output of high-value compounds in *Chlorella vulgaris*. The production strategy of heterotrophic *Chlorella vulgaris* developed in this study could convert sugar in sweet sorghum stems into high-value nutritional products and reduce the culture costs.

## Figures and Tables

**Table 1:** Components of the used media.

Ingredient	Treatment					
	HSE	HSE+N	HSE+M	HSE+N+M	SE+N+M	BG-11+glu
Nitrogen source and mineral						
NaNO_3_ (g/L)		1.500		1.500	1.500	1.500
K_2_HPO_4_ (g/L)			0.040	0.040	0.040	0.040
MgSO_4_·7H_2_O (g/L)			0.075	0.075	0.075	0.075
CaCl_2_·2H_2_O (g/L)			0.036	0.036	0.036	0.036
Citric acid (g/L)			0.006	0.006	0.006	0.006
Ferric ammonium citrate (g/L)			0.006	0.006	0.006	0.006
EDTA·2Na (g/L)			0.001	0.001	0.001	0.001
Na_2_CO_3_ (g/L)			0.020	0.020	0.020	0.020
A5 (mL/L) ^a^			1.000	1.000	1.000	1.000
Carbon source						
Glucose (g/L)						10.000
SE (g/L)					11.400	
HSE (g/L)	10.700	10.700	10.700	10.700		

^a^ The concentrations of H_3_BO_3_, MnCl_2_·4H_2_O, ZnSO_4_, Na_2_MoO_4_, CuSO_4_·5H_2_O, and Co(NO_3_)_2_·6H_2_O in A5 solution were 2.86, 1.81, 0.22, 0.39, 0.08, and 0.05g/L, respectively.

**Table 2:** Components of SE and HSE.

Component	Treatment	
	SE	HSE
Reducing sugar (g/L)	56.75 ± 2.41 ^b^	86.69 ± 2.18 ^a^
Fructose (g/L)	31.28 ± 2.09 ^b^	42.25 ± 0.52 ^a^
Total carbon (g/L)	32.39 ± 0.00 ^b^	34.21 ± 0.00 ^a^
Total organic carbon (g/L)	32.05 ± 0.00 ^b^	34.04 ± 0.00 ^a^
Inorganic carbon (g/L)	0.34± 0.01 ^a^	0.17 ± 0.05 ^b^
Total nitrogen (g/L)	0.18 ± 0.00 ^b^	0.37 ± 0.00 ^a^
Magnesium (mg/L)	62.15 ± 0.15 ^a^	61.40 ± 1.20 ^a^
Potassium (mg/L)	632.30 ± 2.90 ^a^	658.40 ± 8.00 ^a^
Calcium (mg/L)	177.75 ± 7.65 ^a^	170.25 ± 2.55 ^a^
Cuprum (mg/L)	0.00 ± 0.00	0.00 ± 0.00
Manganese (mg/L)	0.00 ± 0.00	0.00 ± 0.00
Iron (mg/L)	0.00 ± 0.00	0.00 ± 0.00
Zine (mg/L)	0.00 ± 0.00	0.00 ± 0.00

Values in the same row with different superscripts (a, b) are significantly different (*p* < 0.05).

**Table 3:** Growth kinetic parameters of the heterotrophic *C. vulgaris*.

Treatment	Generation Time (d)	Biomass Productivity (g/L/d)	Max Biomass (g/L)	Growth Yield (g Biomass/g TOC)
HSE	0.56 ± 0.02 ^a^	0.09 ± 0.00 ^e^	0.54 ± 0.02 ^e^	0.85 ± 0.01 ^d^
HSE+N	0.35 ± 0.01 ^b^	0.25 ± 0.02 ^d^	1.53 ± 0.14 ^d^	1.23 ± 0.03 ^b^
HSE+M	0.55 ± 0.02 ^a^	0.10 ± 0.00 ^e^	0.60 ± 0.02 ^e^	0.79 ± 0.01 ^d^
HSE+N+M	0.29 ± 0.00 ^c^	0.42 ± 0.02 ^b^	2.51 ± 0.15 ^b^	1.30 ± 0.02 ^a^
SE+N+M	0.31 ± 0.01 ^c^	0.34 ± 0.00 ^c^	2.04 ± 0.02 ^c^	1.30 ± 0.02 ^a^
BG-11+glu	0.31 ± 0.01 ^c^	0.52 ± 0.01 ^a^	3.14 ± 0.07 ^a^	1.00 ± 0.03 ^c^

Values in the same row with different superscripts (a–e) are significantly different (*p* < 0.05).

**Table 4:** Nutrients and pigments contents of heterotrophic *C. vulgaris*.

Treatment	Chl-a Content (mg/g)	Carotenoid Content (mg/g)	Lutein Content (mg/g)	Protein Content (g/g)	Lipid Content (g/g)
HSE	11.09 ± 0.36 ^b^	4.56 ± 0.12 ^a^	0.20 ± 0.01 ^c^	-	-
HSE+N	13.33 ± 1.53 ^ab^	5.33 ± 0.49 ^a^	0.55 ± 0.07 ^a^	0.37 ± 0.01 ^d^	0.18 ± 0.01 ^a^
HSE+M	8.59 ± 0.88^c^	3.74 ± 0.27 ^b^	0.25 ± 0.01 ^c^	-	-
HSE+N+M	13.99 ± 0.16 ^a^	5.05 ± 0.15 ^a^	0.40 ± 0.01 ^b^	0.54 ± 0.00 ^b^	0.16 ± 0.00 ^ab^
SE+N+M	13.90 ± 1.03 ^a^	5.07 ± 0.38 ^a^	0.49 ± 0.01 ^ab^	0.58 ± 0.01 ^a^	0.15 ± 0.01 ^b^
BG-11+glu	7.79 ± 0.24 ^c^	3.18 ± 0.13 ^b^	0.15 ± 0.02 ^c^	0.41 ± 0.01 ^c^	0.13 ± 0.00 ^c^

Values in the same row with different superscripts (a–d) are significantly different (*p* < 0.05).

**Table 5:** Cost comparison of media.

Estimate	Unit Price (USD/kg)	HSE+N+M	SE+N+M	BG-11+glu
Biomass yield (kg/10^3^ L medium)		2.175	1.642	2.967
NaNO_3_ amount (kg/10^3^ L medium)	0.537 ^a^	1.500	1.500	1.500
K_2_HPO_4_ amount (kg/10^3^ L medium)	2.385 ^a^	0.040	0.040	0.040
MgSO_4_·7H_2_O amount (kg/10^3^ L medium)	0.146 ^a^	0.075	0.075	0.075
CaCl_2_·2H_2_O amount (kg/10^3^ L medium)	0.188 ^a^	0.036	0.036	0.036
Citric acid amount (kg/10^3^ L medium)	2.086 ^a^	0.006	0.006	0.006
Ferric ammonium citrate amount (kg/10^3^ L medium)	7.452 ^a^	0.006	0.006	0.006
EDTA·2Na amount (kg/10^3^ L medium)	4.322 ^a^	0.001	0.001	0.001
Na_2_CO_3_ amount (kg/10^3^ L medium)	0.402 ^a^	0.020	0.020	0.020
Minerals cost for 1 kg biomass (USD) ^c^		0.454	0.601	0.333
Glucose amount (kg/10^3^ L medium)	0.715 ^a^			10.000
Sweet sorghum stalk amount (kg/10^3^ L medium) ^d^	0.019 ^b^	5.632	6.000	
Carbon source cost for 1 kg biomass (USD)		0.050	0.071	2.411
Sucrase amount (kg/10^3^ L medium)	23.845 ^a^	0.011		
Heating cost of enzymatic hydrolysis (USD)		0.213 ^e^		
Other cost for 1 kg biomass (USD)		0.330	-	-
Total cost for 1 kg biomass (USD)		0.834	0.672	2.744

^a^ The unit price was determined according to the market price in the Chinese circulation market (https://www.rawmex.cn/ (accessed on 18 August 2022)) [47] (USD 1.00 = CNY 6.71, 25 July 2022). ^b^ The unit price was derived from Shi et al. [21] (USD 1.00 = CNY 6.71, 25 July 2022). ^c^ The amount of A5 solution added as a trace element was very low, and its cost was relatively small compared to other minerals. Therefore, its cost was not included. ^d^ The extraction rate of sweet sorghum extract was 76.00% in this study. ^e^ The heating cost was estimated based on electricity consumption.

## Data Availability

The data presented in this study are available upon request from the corresponding author. The data are not publicly available due to privacy.
